# Complete mitochondrial genome of the leaf muntjac (*Muntiacus putaoensis*) and phylogenetics of the genus *Muntiacus*

**DOI:** 10.24272/j.issn.2095-8137.2017.058

**Published:** 2017-09-18

**Authors:** Guo-Gang Li, Ming-Xia Zhang, Kyaw Swa, Kyaw-Win Maung, Rui-Chang Quan

**Affiliations:** ^1^Southeast Asia Biodiversity Research Institute, Chinese Academy of Sciences, Yezin Nay Pyi Taw 05282, Myanmar; ^2^Center for Integrative Conservation, Xishuangbanna Tropical Botanical Garden, Chinese Academy of Sciences, Mengla Yunnan 666303, China; ^3^Hponkan Razi Wildlife Sanctuary Offices, Putao Kachin 01051, Myanmar; ^4^Forest Research Institute, Forest Department Ministry of Environmental Conservation and Forestry, Yezin Nay Pyi Taw 05282, Myanmar

**Keywords:** *Muntiacus*, *Muntiacus putaoensis*, Mitogenome, Phylogenetics

## Abstract

The leaf muntjac (*Muntiacus putaoensis*) is an endemic deer species found in the east trans-Himalayan region. In recent years, population numbers have decreased due to heavy hunting and habitat loss, and little genetic data exists for this species, thus our knowledge of distribution rangs and population sizes likewise remain limited. We obtained mtDNA genes and the complete mitochondrial genome sequence of *M*. *putaoensis* using PCR, followed by direct sequencing. The complete mitogenome sequence was determined as a circular 16 349 bp mitochondrial genome, containing 13 protein-coding genes, two rRNA genes, 22 tRNA genes, and one control region, the gene composition and order of which were similar to most other vertebrates so far reported. Most mitochondrial genes, except for *ND6* and eight tRNAs, were encoded on the heavy strand. The overall base composition of the heavy strand was 33.1% A, 29.3% T, 24.2% C, and 13.4% G, with a strong AT bias of 62.4%. There were seven regions of gene overlap totaling 95 bp and 11 intergenic spacer regions totaling 74 bp. Phylogenetic analyses (ML and BI) among the *Muntiacus* genus based on the sequenced of mitogenome and *ND4L*-*ND4* supported *M*. *putaoensis* as a member of *Muntiacus*, most closely related to *M*. *vuquangensis*. However, when analyses based on cyt *b* included two more muntjacs, *M*. *truongsonensis* was most closely related to *M*. *putaoensis* rather than *M*. *vuquangensis*, and together with *M*. *rooseveltorum*, likely forming a *M*. *rooseveltorum* complex of the species. This study will help in the exploration of the evolutionary history and taxonomic status of the leaf muntjac, as well as its protection as a genetic resource.

## INTRODUCTION

Muntjacs (*Muntiacus* spp., Muntiacinae, Cervidae) are a group of small solitary deer occurring in the forests of Asia. They are of great interest to evolutionary biologists and mammalogists due to their considerable chromosome variations (from 2*n*=6 (*Muntiacus reevesi*) to 2*n*=46 (*M*. *muntjak*)) (Fontana & Rubini, 1990) and discovery of new species in the last decades of the twentieth century, including *M*. *vuquangensis* (Tuoc et al., 1994), *M*. *truongsonensis* (Giao et al., 1998), and *M*. *putaoensis* (Amato et al., 1999; Rabinowitz et al., 1999).

*Muntiacus putaoensis* is the most recently discovered species in this muntjac genus. It is named after the town of Putao, located in the most northern part of Myanmar, which is also its most recognizable reference point (Amato et al., 1999). Compared with other species, it is the smallest muntjac (mean adult body mass 12 kg), and is only half the size of the sympatric *M*. *muntjak* (22–29 kg) (Rabinowitz et al., 1999). Local people call it the 'leaf deer' because it is so small it can be wrapped in a single leaf of *Phrynium capitatum* (Rabinowitz & Khaing, 1998; Rabinowitz et al., 1999). Until recently, information on the distribution range of the species was limited to northern Myanmar. However, it has since been found in the adjoining hill forests of southeastern Tibet, with the local name similar in meaning to its Myanmar name (Choudhury, 2007; Choudhury, 2009; Datta et al., 2003; James et al., 2008). *Muntiacus putaoensis* is endemic to the east trans-Himalayan region (including northern Myanmar and southeastern Tibet, China), the population of the leaf muntjac has decreased dramatically in recent years due to considerable degradation of its natural habitat and heavy hunting pressure (Rabinowitz & Khaing, 1998; Rao et al., 2010; Schaller & Rabinowitz, 2004). This species is listed as data deficient (DD) in the IUCN Red List of Threatened species as there is a lack of certainty about its taxonomy, distribution, population, natural history, and threats (Timmins & Duckworth, 2016). Previously the species was only known to occur along a 70 km east to west stretch in northern Myanmar, the new discovery means a two-fold increase in the total east-west range of this species (James et al., 2008). It also indicates that the extent of its geographic range is still being determined.

The leaf muntjac was characterized and confirmed primarily by its diagnostic mitochondrial DNA (mtDNA) with several partial fragments (Amato et al., 1999). However, its taxonomic status remains controversial as it falls within a group of closely related 'little' muntjacs with strong morphological similarities, including *M*. *rooseveltorum* and *M*. *truongsonensis* (James, et al., 2008; Timmins & Duckworth, 2016). This species is characterized by short thin pedicles, small unbranched antlers, and relatively large preorbital fossa, with both males and females possessing canines (Rabinowitz et al., 1999). Because hunters usually cut off the lower jaw and part of the upper jaw, it is difficult to obtain complete skulls to compare with other muntjacs. In addition, the similarities in morphology also create difficulties when marking comparisons, thus requiring molecular genetic analyses. To date however, genetic data remain scarce, with only partial segments of mtDNA currently sequenced (Amato et al., 1999; James et al., 2008), which are insufficient for confirming the identity of this species or clarifying its phylogenetic relationship relative to other muntjacs.

Here, we sequenced and analyzed the complete mitochondrial genome and two mtDNA segments of *M*. *putaoensis*, the aim of this study was to: 1) provide fundamental genetic data for further conservation genetic studies for this near cryptic mammal; 2) discuss the possible relationships among the genus of *Muntiacus*, especially that of *M*. *putaoensis*, *M*. *rooseveltorum*, and *M*. *truongsonensis*; and, 3) provide further discussion on the evolutionary history of this species.

## MATERIALS AND METHODS

### Sampling and laboratory procedures

**Figure 1:**
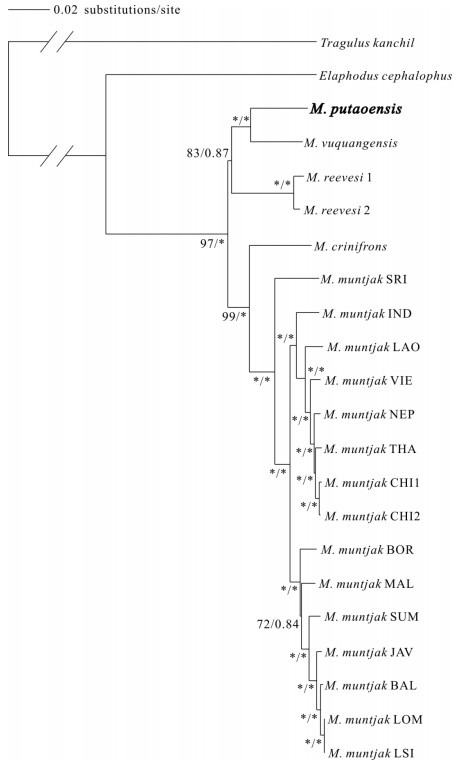
Maximum likelihood (ML) and Bayesian inference (BI) phylogenetic trees (GTR+G model) for *Muntiacus* based on 20 complete genomes from mitochondrial DNA

Seventeen *M*. *putaoensis* specimens were collected from Putao, Kachin state, northern Myanmar in three field trips (December 2015, December 2016 to January 2017, and May 2017) (Figure 1; Supplementary Table S1). Three skin samples were preserved in 95% ethanol, and 14 dry meat samples were preserved using silica gel for subsequent analyses. Voucher specimens were deposited in the Southeast Asia Biodiversity Research Institute, Chinese Academy of Sciences, in Nay Pyi Taw, Myanmar (Supplementary Table S1).

Total genomic DNA was extracted from tissue using a DNeasy Blood & Tissue Kit (Qiagen, Shanghai, China). The master mixture for polymerase chain reaction (PCR) contained approximately 100 ng of template DNA, 1 μL (10 pmol) of each primer, 5 μL of 10× reaction buffer, 2 μL of dNTPs (2.5 mmol/L of each), and 2.0 U of Taq DNA polymerase, in a total volume of 50 μL. Reactions were carried out on a Veriti Thermal Cycler (Applied Biosystems, Carlsbad, CA, USA) and always included a negative control. The *M*. *putaoensis* mtDNA segments (*ND4L*-*ND4* and cyt *b*) were amplified using PCR with universal primers from previous studies (Irwin et al., 1991; Wang & Lan, 2000). After visualization of the fragments using 1% agarose gel, the PCR products were sequenced from both ends using an ABI PRISM 3700 sequencing system with using the same primers as for PCR (Beijing Tianyi Huiyuan Bioscience and Technology Incorporation, Beijing, China).

To obtain the whole mitogenomic sequence of *M*. *putaoensis*, we designed 16 pairs of primers based on previous studies (Hassanin et al., 2012; Larkin et al., 2007; Martins et al., 2017; Shi et al., 2003; Wu & Fang, 2005; Zhang et al., 2004a, b). The primer sequences and approximate lengths of the amplified fragments from the whole genome are listed in Supplementary Table S2. All the primers were synthesized by Beijing Tianyi Huiyuan Bioscience and Technology Incorporation (Beijing, China).

### Sequence analyses

Two mitochondrial DNA segments sequences were edited using the program DNASTAR 5.0 (DNASTAR Inc.), and were aligned using the CLUSTALW algorithm implemented in MEGA 5.05 with default parameters (Larkin et al., 2007; Tamura et al., 2011). Positions of ambiguous alignment and those from variable length intergenic regions were excluded from further analyses. Identical haplotypes were collapsed using DNASP 5.1 (Librado & Rozas, 2009). The base composition of the mitogenomic sequence was analyzed using MEGA 5.05 (Tamura et al., 2011). We annotated the genome sequence using DOGMA (Wyman et al., 2004).

### Phylogenetic analyses

Phylogenies of the two mtDNA segments and mitogenome were constructed using maximum likelihood (ML) implemented in PHYML 3.0 (Guindon et al., 2010). Bayesian inference (BI) was implemented in MRBAYES 3.2.1 (Ronquist et al., 2012) using different parameter estimates for the two mtDNA segments and mitogenome. The most appropriate nucleotide substitution models for the two segments and genome were selected using the Akaike Information Criterion in JMODELTEST 2.1.4 (Darriba et al., 2012). To assess the statistical significance of the hypothesized lineages, bootstrap analysis with 200 replicates was used for the ML analyses, with other settings set to default. The posterior distributions were obtained by Markov Chain Monte Carlo (MCMC) analysis with one cold chain and three heated chains. Samples of the trees and parameters were drawn every 100 steps from a total of one million MCMC generations. Three additional runs were conducted, beginning with random trees. A 50% majority rule consensus of the post-burn (using a burn-in of 25%) for all generations was computed for all four runs. *Elaphodus cephalophus* and *Tragulus kanchil* were chosen as outgroups to root the tree.

## RESULTS AND DISCUSSION

### Sequence data and genome content

Cyt *b* sequences with a total length 1 140 bp were obtained from all 17 *M*. *putaoensis* individuals with four variable sites. We obtained 1 844 bp of unambiguous sequences (*ND4L*-*ND4*) from 13 individuals, which contained the complete sequences of NADH dehydrogenase subunit 4L (*ND4L*), NADH dehydrogenase subunit 4 (*ND4*), tRNA^Ser^, and tRNA^His^ genes, and a partial sequence of the tRNA^Leu^ gene, with 15 variable sites and six parsimony-informative sites. We detected three cyt *b* haplotypes and eight *ND4L*-*ND4* haplotypes separately (GenBank accession Nos. MF737179– MF737190, Supplementary Table S1).

The complete mitochondrial genome features of *M*. *putaoensis* were identical to those of most other muntjac deer (e.g. Hassanin et al., 2012; Shi et al., 2003; Wu & Fang, 2005; Zhang et al., 2004a, b). The complete mitogenome sequence was a circular molecule 16 349 bp in length, and included 13 protein-coding genes (*ATP6*, *ATP8*, *COⅠ*-*Ⅲ*, cyt *b*, *ND1*-*6*, and *ND4L*), two rRNA genes (12S and 16S rRNA), 22 tRNA genes, and one non-coding control region (D-loop), most of which were encoded on the heavy strand, except for *ND6* and eight tRNAs (tRNA^Gln^, tRNA^Ala^, tRNA^Asn^, tRNA^Cys^, tRNA^Tyr^, tRNA^Ser^, tRNA^Glu^, and tRNA^Pro^). Notably, gene overlap and separation, common characteristics found in other vertebrate mitochondrial genomes, were also observed in the *M*. *putaoensis* mitogenome. There were seven regions of gene overlap totaling 95 bp (varying from 1 to 40 bp) and 11 intergenic spacer regions totaling 74 bp (varying from 1 to 32 bp) (Table 1). The overall base composition of the heavy strand was 33.1% A, 29.3% T, 24.2% C, and 13.4% G with a strong AT bias of 62.4%. Nearly all 13 protein-coding genes started with the common initiation codon ATG, whereas *ND2*, *ND3*, and *ND5* started with ATA. In addition, 10 protein-coding genes shared the complete stop codons TAA or TAG (TAA for *ND1*, *COⅠ*, *COⅡ*, *ATP6*, *ND4L*, *ND5*, and *ND6*, and TAG for *ND2*, *ATP8*, and *ND3*), whereas the remaining three protein-coding genes (*ND4*, *COⅢ* and cyt *b*) possessed incomplete stop codons with a terminal T or TA. The 22 tRNA genes were interspersed along the genome, with lengths varying from 60 to 75 bp. The lengths of 12S rRNA and 16S rRNA were 958 and 1 568 bp, respectively, located between tRNA^Phe^ and tRNA^Leu (UUR)^ and separated by tRNA^Val^. The D-loop region was located between tRNA^Pro^ and tRNA^Phe^, and was 914 bp in length. The base composition of the D-loop was A-29.9%, T-31.9%, C-23.1%, and G-15.1%, reﬂecting a strong feature in A and T (A+T=61.8%).

**Table 1:** Characteristics of the mitochondrial genome of *Muntiacus putaoensis*

Codon							
Gene/Element	From	To	Length (bp)	Start	Stop	Intergenic nucleotides^*^	Strand^†^
tRNA-Phe	1	69	69				H
12S rRNA	70	1027	958				H
tRNA-Val	1028	1095	68				H
16S rRNA	1096	2663	1568			1	H
tRNA-Leu (UUR)	2665	2739	75			2	H
*ND1*	2742	3698	957	ATG	TAA	-1	H
tRNA-Ile	3698	3766	69			-3	H
tRNA-Gln	3764	3835	72			2	L
tRNA-Met	3838	3906	69				H
*ND2*	3907	4950	1044	ATA	TAG	-2	H
tRNA-Trp	4949	5016	68			1	H
tRNA-Ala	5018	5086	69			1	L
tRNA-Asn	5088	5160	73			32	L
tRNA-Cys	5193	5260	68				L
tRNA-Tyr	5261	5329	69			1	L
*COⅠ*	5331	6875	1545	ATG	TAA	-3	H
tRNA-Ser (UCN)	6873	6941	69			7	L
tRNA-Asp	6949	7016	68			1	H
*COⅡ*	7018	7701	684	ATG	TAA	3	H
tRNA-Lys	7705	7771	67			1	H
*ATP8*	7773	7973	201	ATG	TAG	-40	H
*ATP6*	7934	8614	681	ATG	TAA	-1	H
*COⅢ*	8614	9398	785	ATG	TA-	-1	H
tRNA-Gly	9398	9466	69			9	H
*ND3*	9476	9823	348	ATA	TAG	-10	H
tRNA-Arg	9814	9882	69				H
*ND4L*	9883	10179	297	ATG	TAA	-7	H
*ND4*	10173	11550	1378	ATG	T--		H
tRNA-His	11551	11619	69				H
tRNA-Ser (AGY)	11620	11679	60			1	H
tRNA-Leu (CUN)	11681	11750	70			-9	H
*ND5*	11742	13571	1830	ATA	TAA	-17	H
*ND6*	13555	14082	528	ATG	TAA		L
tRNA-Glu	14083	14151	69			4	L
cyt *b*	14156	15290	1135	ATG	T--	8	H
tRNA-Thr	15299	15368	70			-1	H
tRNA-Pro	15368	15435	68				L
D-loop	15436	16349	914				-
^*^Numbers correspond to the nucleotides separating different genes. Negative numbers indicate overlapping nucleotides between adjacent genes; ^†^H and L denote heavy and light strands, respectively.							

### Phylogenetic analysis

**Figure 2:**
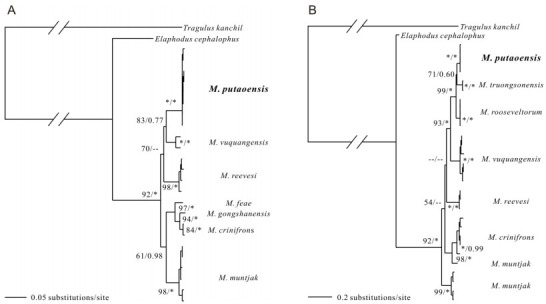
Maximum likelihood (ML) and Bayesian inference (BI) phylogenetic trees (GTR + G model) for *Muntiacus* based on *ND4L*-*ND4* genes (A) and cyt *b* gene (B)

The phylogeny of the genus *Muntiacus* has long been debated (James et al., 2008; Timmins & Duckworth, 2016). Here, we conducted a phylogenetic analysis using complete mitochondrial genomes from five muntjac species (others have not yet been sequenced for mitochondrial genomes) (Figure 1). Additional sequences and species were included in phylogenetic analysis based on *ND4L*-*ND4* (Figure 2A) and cyt *b* (Figure 2B). The ML and BI analyses recovered the same tree topologies (Figure 1 and 2).

The three different level sequences obtained in this study (i.e., complete mitogenome, *ND4L*-*ND4*, and cyt *b*) provide insight into the taxonomic status of *Muntiacus*.

Based on the complete genomes (Figure 1), the data showed two major clades within the genus of *Muntiacus*, one of which included *M*. *putaoensis*, *M*. *vuquangensis* and *M*. *reevesi*, and the other *M*. *crinifrons* and *M*. *muntjak*. In the latter clade, *M*. *crinifrons* was positioned on a distinct branch as sister to *M*. *muntjak*. Within *M*. *muntjak*, three phylogenetically and geographically distinct groups were previously identified by Martins et al. (2017). We determined the leaf muntjac (*M*. *putaoensis*) to be a member of genus *Muntiacus*, most closely related to *M*. *vuquangensis*, with *M*. *reevesi* as their sister species. However, as genomic data for other muntjacs is still unavailable, further genomic research on more species may illuminate different relationships within this taxon.

Previous phylogenetic information has been provided for the genus *Muntiacus* from mtDNA genes (Amato et al., 1999; Wang & Lan, 2000). Based on eight haplotypes of *M*. *putaoensis* obtained in this study, combined with the *ND4L*-*ND4* sequences of six other muntjacs downloaded from GenBank, our phylogenetic results showed *M*. *putaoensis* as a member of genus *Muntiacus* (Figure 2A), consistent with the above mitochondrial genome findings.

However, when *M*. *truongsonensis* and *M*. *rooseveltorm* were included in analyses based on cyt *b* (Figure 2B), we found that *M*. *truongsonensis*, rather than *M.* *vuquangensis*, was most closely related to *M*. *putaoensis*. Furthermore, we observed only weak support (bootstrap supports for ML/posterior probability in BI=71/0.60; Figure 2B) among these three species, and that they all likely belonged to the *M*. *rooseveltorum* complex of species. This result is consistent with previous morphological studies (James et al., 2008; Timmins & Duckworth, 2016).

Complete mitogenome data of *M*. *putaoensis* would be fundamental to research phylogentic relationship and conservation genetics of muntjac genus. As the current conclusion is based on analysis of only a few mtDNA fragments, comparison of longer sequences or genomes will be more convincing. More studies based on genome are required to better illuminate wholesome view of phylogenetics and evolution in this extraordinary taxon.

## ACKNOWLEDGEMENTS

The authors sincerely thank Prof. Shu-Qiang Li and Dr. Yu Song for their support with the laboratory work. We also thank Mr. Kyaw Kyaw Han and Shuyin Huang for sample collection, thank Ms. Nan Sun and Alice Hughes for proofreading the manuscript. This work was possible because of the support from the Forest Research Institute, Hponkan Razi Wildlife Sanctuary and Hkakaborazi National Park Offices, Myanmar.
